# Supplementary material for: The Patients’ Long-Term Insight into Day-to-Day Functioning After Establishing the Functional Seizures Diagnosis
Source: Brain Sci. 2025 Jan 26;15(2):120. doi: 10.3390/brainsci15020120 (PMC11853039; doi:10.3390/brainsci15020120)
Supplement: Supplementary file 1 [file brainsci-15-00120-s001.zip › brainsci-3375678-supplementary.pdf]

Supplementary Table S1. List of all variables used and results of univariate logistic regression on good QoL.

Variables	Odds Ratio	Lower 95% CI	Upper 95% CI	P-Value
Sex_Male	0,72727273	0,204055068	2,592072938	0,62334203
Absence of comorbidity	2,33333333	0,7576357	7,186098075	0,13984261
Neurological comorbidity	0,41304348	0,105268806	1,620659738	0,2048932
The induction test	1,42352941	0,537742394	3,76841404	0,4770945
Absence of psychiatric testing	0,46245059	0,116727477	1,832135463	0,27221453
Age at FS onset	0,95692163	0,919993642	0,995331876	0,02830501
Perceiving oneself as mentally changed	0,16000002	0,019299007	1,32649342	0,08947339
Perception_of_disease	0,72727273	0,204055068	2,592072938	0,62334203
Disease_severity	4,55384615	1,655733031	12,524673	0,00331553
Belief in treatment's positive effects	3,35802469	1,243873511	9,065495588	0,01681659
Diagnosis_understanding	1,30555556	0,204141833	8,349466085	0,7782238
Diagnosis provided in discharge summary only	0,62666667	0,061916212	6,342621751	0,69229855
Diagnosis explained with psychiatric referral	1,57575758	0,541393558	4,586334467	0,40413164
Duration_of_disease	0,9832839	0,926879794	1,043120404	0,57595238
Change of frequency of FS	0.00	0	100	0,99181387
FS cessation	6,28571429	1,990627415	19,84811612	0,00172716
Duration of the FS	1,50678733	0,540226663	4,202695306	0,43339755
Severity_of_seizure	1,01973684	0,384686887	2,703141859	0,96865529
Presence of body shaking	2	0,750622386	5,328911147	0,16565693
Presence of shaking in body parts	0,6446281	0,18320352	2,268217259	0,49393689
Dystonia as a manifestation	0.00	0	100	0,99232571
Non responding as a manifestation	0,73109244	0,273296388	1,955738072	0,53269652
Pseudosleep as a manifestation	0,95833333	0,163610947	5,613333294	0,9623623
Hypermotor seizure as a manifestation	0,61111111	0,114370058	3,265337078	0,56462713
Sensory symptom as a manifestation	6,39130435	0,630111125	64,82788451	0,11658519
Pseudoaura	0,79545455	0,302294769	2,093148805	0,64294077
Changing the manifestation of FS	0,95833333	0,163610947	5,613333294	0,9623623
Nonphysiologic movements	0,67765568	0,211980004	2,166323283	0,5116588
Communication during FS	0,72727273	0,204055068	2,592072938	0,62334203
Seizure clusters	1,77777778	0,601657753	5,252976154	0,29793449
Intervention stoping the FS	1,125	0,412645221	3,067102045	0,81795524
Taking the treatment regularly	2,73809524	0,666816083	11,24322842	0,16220778
Neurologist check – ups	0,42328042	0,136261799	1,314868277	0,13710767
Hospital stayings	0.00	0	100	0,99232571
Psychotherapy	0,73109244	0,273296388	1,955738072	0,53269652
Depression symptoms	0,10227273	0,030442867	0,343584949	0,00022607
No fear of the next FS	3,375	1,233112296	9,237297397	0,01788912

Fear of injury	0,45098039	0,169108899	1,202676592	0,11155729
Fear of therapy side effects	0,27717391	0,072440318	1,060533418	0,06090899
Reduction of the seizure number	2,25	0,827336106	6,119036706	0,11213504
Current age of life	0,93779795	0,897169198	0,980266586	0,00448303
